# Korean botulinum toxins

**DOI:** 10.1007/s00702-025-03076-x

**Published:** 2026-01-31

**Authors:** Dirk Dressler, Nayoung Kim, Eric A. Johnson, Fereshte Adib Saberi

**Affiliations:** 1 Movement Disorders Section, Department of Neurology, Hannover Medical School, Carl-Neuberg-Str. 1, D-30625 Hannover, Germany; 2 Botulinum Toxin Research Center, Tongji University Medical School, Shanghai, China; 3 Stellar-B, Seoul, Republic of Korea; 4 Department of Bacteriology, University of Wisconsin-Madison, Madison, WI USA; 5 IAB - Interdisciplinary Working Group for Movement Disorders, Hamburg, Germany

**Keywords:** Botulinum toxin, Drugs, Manufacturers, Korea, Therapeutic use, Aesthetic use

## Abstract

Botulinum toxin (BT) is used therapeutically since the late 1980s. For many years, BT drugs were provided by a small group of manufacturers from Europe and the United States. As BT’s use for aesthetic purposes surged, numerous new manufacturers entered the field, particularly from Korea. We want to give an overview about the rapidly expanding and diversifying BT landscape in Korea. Altogether, there are 15 Korean manufacturers of 19 BT drugs registered in Korea, mainly for aesthetic indications. There are 2 Class 1 drugs from 2 Korean manufacturers registered in Korea for export, domestic use and registered in the USA and Europe. These are PrabotulinumtoxinA (Daewoong/Evolus) and LetibotulinumtoxinA (Hugel/Croma Pharma). There are 12 Class 2 drugs from 10 Korean manufacturers registered in Korea for export and domestic use, including Neuronox^®^, the first Korean BT drug registered in 2006, NivobotulinumtoxinA/Innotox^®^, the world-wide first liquid BT type A preparation and Coretox^®^, the world-wide second BT drug without complexing proteins. Innotox^®^ and Toxsta^®^ are currently performing clinical studies in the USA. Additionally, there are 5 Class 3 BT drugs from 5 Korean manufacturers registered in Korea for export use only. 5 Korean manufacturers have 5 drug projects in Korea in advanced development phases. With this, Korea is now the country with the worldwide largest number of BT manufacturers and BT drugs.

## Introduction

Botulinum toxin (BT) type A is used therapeutically since the late 1980s. Its aesthetic use developed later. For many years, BT drugs were provided by a small group of manufacturers including Allergan from the United States, Ipsen from England and France and later on by Merz from Germany. In the late 1990s, Lanzhou Institute Institute of Biological Products produced a Chinese BT drug (Dressler et al. 2021), but did not introduce it in the North American and European markets. Elan from the USA tried to develop a BT type B drug, but failed to gain relevant market share (Dressler and Benecke 2003; Dressler and Bigalke 2004). As BT’s use for aesthetic purposes surged, now accounting for about half of all BT sales, numerous new manufacturers entered the field, particularly from Asia. Amongst all countries worldwide, Korea is now the country with the largest number of BT manufacturers.

We want to give an overview about rapidly expanding and diversifying BT landscape in Korea.

## Methods

This review is based on publicly available information including company presentations on the internet, at conferences and as press releases, on general media and internet information and public registers including Drug Approval Reports provided by the Korean Ministry of Food and Drug Safety. The information is accurate as of April 1st, 2024. It is strongly advised to update all information if necessary, as the field is extremely rapidly changing.

This review is also based on background discussions, abstractions and insight gained by several years of consultancies to many players in the field (DD), of course under strict maintenance of confidentiality of privileged information.

### Overview

Reviewing the Korean BT market is challenging for several reasons: It is extremely rapidly expanding with large numbers of companies being involved. Most of these companies are new companies and usually they are privately held, so that public reporting is scarce. Often, they are collaborating with each other, so that responsibilities within these partnerships are sometimes difficult to attribute.

The Korean domestic market is believed to be US$194.1 million in 2023 with a massive dominance of aesthetic use (Grandviewresearch 2024). Foreign BT drugs registered in Korea include OnabotulinumtoxinA, AbobotulinumtoxinA and IncobotulinumtoxinA. Their pricing is similar to that of other industrialised countries with prices for OnabotulinumtoxinA and IncobotulinumtoxinA of around US$ 220–370 per 100MU vial. With its considerable size and robust prices, the Korean market attracted large numbers of Korean competitors producing what now is often referred to as ‘K-Botox’. However, with this increasing competition, prices for K-Botox soon dwindled. They are now around US$100 to 180 per 100MU vial, if products also have USA registrations. K-Botox without USA registration may be sold for around US$50 per 100MU vial. Because of this, Korean BT manufacturers have been increasingly reaching out to foreign markets, especially in Asia. There is now massive exportation of K-Botox to those markets. A relatively new export market for K-Botox is China with its huge market size and relatively easy market access, especially for Korean companies. The most attractive market, however, remains the USA market, with annual sales of US$4.67 billion in 2023 (Fortunebusinessinsights 2024) and an official Botox^®^ price of around US$650 for a 100MU vial. It covers 42.1% of the global BT market of US$11.1 billion in 2023 (Fortunebusinessinsights 2024).

### General comments

Korean BT drugs can be classified according to their registration status. Class 1 drugs are produced and registered in Korea for export and for domestic use and they are registered in the USA by the Food and Drug Administration (FDA) and/or the European Union by the European Medicines Agency (EMA). Class 2 drugs are produced and registered in Korea for export and for domestic use. They have no registration in the USA and/or Europe. Class 3 drugs are produced and registered in Korea for export use only.

There are 15 Korean manufacturers providing 19 BT drugs produced and registered in Korea and 5 Korean manufacturers working on 5 BT drug development projects in Korea. More Korean companies are distribution partners and development partners of these primary BT manufacturers.

### Class 1 drugs

As shown in Table 1, there are only two Class 1 drugs: Prabotulinumtoxin A manufactured by Daewoong and LetibotulinumtoxinA manufactured by Hugel. Both are freeze-dried BT type A drugs containing complexing proteins. In the United States of America and in the European Union, both BT drugs are registered for treatment of glabella lines, which is the largest aesthetic indication of BT drugs. Registration with the FDA and EMA indicates, that both manufacturers have mastered the specific manufacturing requirements described in their Good Manufacturing Practice (GMP) regulations, which are usually a hurdle for manufacturers looking to introduce their products to these markets.

**Table 1:** Class 1 botulinum toxin drugs manufactured in Korea

Generic Name	Trade Name	Manufacturer and Partners	Features	USP	Registration	Coverage	Additional Trade Names
LetibotulinumtoxinA	Botulax^®^	Hugel	BT-A-C FD A/T		Korea 2009	D/E	Aestox^®^
	Letybo^®^	Hugel Croma-Pharma	BT-A-C FD A		Europe 2022, USA 2024		
PrabotulinumtoxinA	Nabota^®^	Daewoong	BT-A-C FD A/T		Korea 2013	D/E	
	Jeuveau^®^	Daewoong Evolus	BT-A-C FD A/T	first Korean BT in USA	USA 2019		
	Nuceiva^®^	Daewoong Evolus	BT-A-C FD A/T	first Korean BT in Europe	Europe 2020		

BT-A-C botulinum toxin type A containing complexing proteins, BT-A botulinum toxin type A free of complexing proteins, FD freeze dried (lyophilisation), VD vacuum-dried, A aesthetic indication, T therapeutic indication, D Korean domestic registration, E Korean export registration, USP Unique Selling Point (as claimed by the manufacturer), Korea Republic of Korea, USA United States of America

*Daewoong Pharmaceuticals*: This is a Seoul based Korean pharmaceutical company with about 1300 employees, 2022 revenues of about US$1.1 billion and a current market capitalisation of about US$1.2 billion. It was founded in 1945. It was the third manufacturer producing BT drugs in Korea. Its BT drug PrabotulinumtoxinA was introduced in Korea in 2013. It is marketed in aesthetic and therapeutic indications. Based on a partnership with Evolus, it was the first Korean BT drug reaching the USA in 2019 under the brand name of Jeuveau^®^ and the first Korean BT drug reaching Europe in 2021 under the brand name of Nuceiva^®^. Daewoong was entangled in a long-standing legal battle with Medytox over the ownership of their breeding strain and manufacturing know-how. After rulings in favour of Medytox by Korean courts and the USA International Trade Commission, the case was finally settled between both parties.

*Hugel*: This is a biopharmaceutical company focussed on aesthetic medicine including BT and hyaluronic acid fillers. It is based in Chuncheon, Korea, has approximately 650 employees, 2023 revenues of about US$244 million and a current market capitalisation of about US$2.1 billion. It was founded 2001. It was the second manufacturer of BT drugs in Korea. Its BT drug LetibotulinumtoxinA was introduced in Korea in 2009 as Botulax^®^. In partnership with Croma Pharma, it was registered in Europe in 2022 and in the USA in 2024 under the brand name Letybo^®^. In Korea and the USA, it is registered for aesthetic and therapeutic indications, in Europe for aesthetic indications only.

### Class 2 drugs

As shown in Table 2, there are 12 class 2 drugs produced by 10 Korean manufacturers. Two of them, NivobotulinumtoxinA/Innotox^®^ manufactured by Medytox and developed together with Abbvie-Allergan and Toxsta^®^ manufactured by Jetema, are performing clinical trials in the USA, indicating that they must use FDA-GMP manufacturing facilities. All 12 class 2 drugs use BT type A containing complexing proteins. NivobotulinumtoxinA (Innotox^®^ ) comes as a liquid preparation, all others use BT powder. Most are registered for aesthetic indications, some for aesthetic and therapeutic indications. Unique selling points, as claimed by the manufacturers, include ‘world-wide first liquid preparation’ (NivobotulinumtoxinA/Innotox^®^), ‘first Korean BT’ (Neuronox^®^), ‘animal free product’ (Coretox^®^, Newlux^®^), ‘free of complexing proteins’ (Coretox^®^) and using ATCC3502 and ‘superior vacuum drying’ (Biennox^®^).

**Table 2:** Class 2 botulinum toxin drugs manufactured in Korea

Generic Name	Trade Name	Manufacturer and Partners	Features	USP	Registration	Coverage	Additional Trade Names
	Neuronox^®^	Medytox	BT-A-C FD A + T	first Korean BT	Korea 2006	D/E	Meditoxin^®^ Siax^®^ Botulift^®^ Cunox^®^ Acebloc^®^
NivobotulinumtoxinA	Innotox^®^	Medytox	BT-A-C A + T	world-wide first liquid preparation	Korea 2013	D/E	
	Coretox^®^	Medytox	BT-A FD A + T	animal free preparation free of complexing proteins	Korea 2016	D/E	
	Hutox^®^ Liztox^®^	Huons BioPharma	BT-A-C FD A		Korea 2016	D/E	Mbtox^®^
	WonderTox^®^	Chong Kun Dang Pharm (CKD) Huons BioPharma	BT-A-C A		Korea 2019	D/E	
	Lientox^®^	Pharma Research Bio	A		Korea 2019	D/E	Rientox^®^ Re N Tox^®^
	Hitox^®^	BMI Korea	A		Korea 2020	D/E	Mulex^®^
	Inibo^®^	Inibo	BT-A-C A		Korea 2020	D/E	
	Bienox^®^	BNC Korea	BT-C VD	VD	Korea 2020	D/E	
	Vivitoxin^®^	Humedix Huons BioPharma	BT-A-C		Korea 2021	D/E	
	Atoxin^®^	Daewoong	A/T		Korea 2022	D/E	
	Newlux^®^	Numeco Medytox	BT-A-C FD A	animal free	Korea 2023	D/E	

BT-A-C botulinum toxin type A containing complexing proteins, BT-A botulinum toxin type A free of complexing proteins, FD freeze dried (lyophilisation), VD vacuum-dried, A aesthetic indication, T therapeutic indication, D Korean domestic registration, E Korean export registration, USP Unique Selling Point (as claimed by the manufacturer), Korea Republic of Korea, USA United States of America

*Medytox*: This is a biopharmaceutical company focussed on BT and fillers. It is based in Seoul, Korea, has approximately 760 employees, 2023 revenues of about US$165 million and a current market capitalisation of US$960 million. It was founded in 2000 and now operates several plants in Korea. It produced the first Korean BT drug in 2006 under the name of Neuronox^®^ (Meditoxin^®^, Siax^®^, Botulift^®^, Cunox^®^, Acebloc^®^). It is a freeze-dried, BT type A drug containing complexing proteins. It is registered for aesthetic and therapeutic indications. In parallel, Medytox developed other BT drugs with different features: Innotox^®^ was the world-wide first liquid BT type A drug. It was first registered in Korea in 2013. Coretox^®^ is the world-wide second BT drug using BT without complexing proteins. It also does not contain human or animal proteins.

*Huons Biopharma*: This is a biomedical company producing BT drugs. It was spun off Huons Global in 2021 and is based in Seongnam-si, Korea. It registered Hutox^®^ or Liztox^®^, a freeze dried BT type A drug containing complexing proteins in Korea in 2019. It is registered for aesthetic indications. An expansion into Europe is planned in partnership with HaematoPharm from Berlin, Germany. HU-045, a BT type A drug without complexing proteins, is in clinical development.

*Chong Kun Dang Pharm*: This is a Korean pharmaceutical company based in Seoul, Korea. It was founded in 1941, has about 2400 employees, 2023 revenues of about US$ 1.15 billion and a current market cap of US$745 million. In 2019, it registered WonderTox^®^ in Korea. Several distributors claim US FDA registration for WonderTox^®^ on the Internet. However, this could not be verified.

*Numeco*: This is a Korean company based in Seoul. It is affiliated with Medytox and has produced a freeze-dried BT type A drug containing complexing proteins for aesthetic use under the brand name Newlux^®^. It does not contain human or veterinary proteins like human serum albumin. It is manufactured at Medytox’s plant #3.

### Class 3 drugs

As shown in Table 3, there are 5 Class 3 BT drugs manufactured by 5 Korean manufacturers. They seem to be BT type A drugs containing complexing proteins and their indications are mostly aesthetic. Toxsta^®^ manufactured by Jetema claims the use of a ‘superior strain used by European manufacturers’.

**Table 3:** Class 3 botulinum toxin drugs manufactured in Korea

Generic Name	Trade Name	Manufacturer and Partners	Features	USP	Registration	Coverage	Additional Trade Names
	Toxsta^®^	Jetema	BT-A-C A	use of superior ATCC3502 breeding strain	Korea 2020	E	Jetema-The-Toxin^®^
	Toxnine^®^	Medica Korea	A		Korea 2020	E	
	Tyemvers^®^	CKD Bio	A		Korea 2022	E	
	BTSA^®^	Protox	A		Korea 2020	E	Protoxin^®^
	BotaOne^®^	Genetox			Korea 2022	E	

BT-A-C botulinum toxin type A containing complexing proteins, BT-A botulinum toxin type A free of complexing proteins, FD freeze dried (lyophilisation), VD vacuum-dried, A aesthetic indication, T therapeutic indication, D Korean domestic registration, E Korean export registration, USP Unique Selling Point (as claimed by the manufacturer), Korea Republic of Korea, USA United States of America

### BT drug development projects

As shown in Table 4, there are currently 5 BT drug development projects in Korea. 4 are based upon BT type A containing complexing proteins, 1 on BT without complexing proteins. All are targeted at aesthetic indications. NeoTox^®^ and Metox^®^ claim application of superior vacuum drying. NeoTox^®^ also claims advantages of using a Hall A strain from a ‘European National Organization’. What this is and why this matters, remains unclear. Whether they actually have USA-FDA-GMP and EMA-GMP, could not be verified.

**Table 4:** Botulinum toxin drugs development projects in Korea

Generic Name	Trade Name	Manufacturer and Partners	Features	USP	Registration	Coverage	Additional Trade Names
	NeoTox^®^	Neo Genesis	BT-A-C VD A	genuine Hall A strain certified by a European National Organization			
	Zerotox^®^	Double JHolding	BT-A-C A				
	ATGC-110^®^	ATGC Menarini-Relife	BT-A-C A				
	Metox^®^	Maypharm	BT-A-C VD A	VD			
	HU-045	Huons BioPharma	BT-A				

BT-A-C botulinum toxin type A containing complexing proteins, BT-A botulinum toxin type A free of complexing proteins, FD freeze dried (lyophilisation), VD vacuum-dried, A aesthetic indication, T therapeutic indication, D Korean domestic registration, E Korean export registration, USP Unique Selling Point (as claimed by the manufacturer), Korea Republic of Korea, *USA* United States of America

## Data Availability

No datasets were generated or analysed during the current study.
